# Efficacy of Honey as A Moisturizing Ingredient in Cosmetic Formulations: A Systematic Review of Clinical Trials

**DOI:** 10.1111/jocd.71039

**Published:** 2026-07-10

**Authors:** Pedro Henrique Fonseca Veloso, Vanessa De Andrade Royo

**Affiliations:** ^1^ Laboratory of Natural Products, Department of General Biology State University of Montes Claros Montes Claros MG Brazil

**Keywords:** clinical efficacy, honey, skin hydration, topical formulations, wound healing

## Abstract

**Background:**

Honey is a viscous, sweet, and aromatic substance produced by bees from floral nectar or plant secretions. Recognized by the International Nomenclature of Cosmetic Ingredients (INCI) as a cosmetic ingredient with humectant, emollient, and moisturizing properties, honey has been increasingly investigated in cosmetic formulations due to its beneficial effects on the skin. Aims: This study aimed to systematically review published articles on the efficacy of honey as a cosmetic ingredient with moisturizing properties in clinical trials.

**Methods:**

The PRISMA (Preferred Reporting Items for Systematic Reviews and Meta‐Analyses) flow diagram was used as a tool for study inclusion and exclusion. Articles were retrieved from Scopus, PubMed, Web of Science, Embase, and Cochrane Library databases up to April 2025. The search terms included “honey”, “skin”, “dermis”, “epidermis”, “hydration”, “moisturization”, “moisturizing”, “water retention”, “efficacy”, “effectiveness”, “clinical effect”, and “outcome”.

**Results:**

A total of five studies were included in this review. Three studies reported relevant findings regarding honey‐based cosmetic formulations, particularly in terms of skin hydration and wound healing efficacy, while two studies showed non‐significant results. The analysis suggests that the moisturizing potential of honey may be associated with its botanical origin, especially the content of reducing sugars, which enhance water retention in the skin. Furthermore, other constituents, such as amino acids and antioxidant compounds, contribute to its moisturizing and regenerative properties.

**Conclusions:**

Therefore, honey represents a promising alternative for the development of moisturizing cosmetic formulations, with advantages related to its natural origin and low risk of adverse effects.

## 1 Introduction

Honey is a viscous, sweet, and aromatic solution produced by bees from floral resources or secretions of living plant parts [1]. It is composed of amino acids, proteins, organic acids, vitamins, minerals, phytoconstituents, and predominantly sugars [2, 3]. In addition to its nutritional value, honey is recognized as a cosmetic ingredient by the International Nomenclature of Cosmetic Ingredients (INCI) under the descriptors “mel” or “honey” (CAS No. 8028‐66‐8), classified as a moisturizer, humectant, and emollient [4]. Its derivatives include “Honey Extract” (CAS No. 91052–92‐5/8028‐66‐8), categorized as a moisturizer, and “Hydrogenated Honey” (CAS No. 223705–79‐1), recognized as a humectant and skin‐conditioning agent [5].

The complex mixture of compounds and their physicochemical properties make honey capable of promoting skin hydration [6]. Among its applications, medical‐grade honey has been used as an alternative treatment for infections and wounds, aiding wound healing, re‐epithelialization, angiogenesis, stimulation of skin cells, and immune response activation [7, 8]. Additionally, honey has been reported to stimulate lymphocytes and phagocytes [9], induce epithelial repair through molecular markers [10], and trigger epithelial–mesenchymal transition in keratinocytes, thereby promoting the formation of a protective skin barrier [11].

Aiming to preserve and enhance skin hydration, cosmetic products act through various mechanisms and are formulated with a range of ingredients capable of producing dermal or transdermal effects, contributing to the maintenance of skin quality and elasticity. Moisturizers, therefore, play a fundamental role in both the treatment of dermatological conditions and in preserving skin health [12]. Furthermore, the demand for natural ingredients or plant‐derived dermocosmetics has significantly increased in recent years, driven by their enhanced efficacy and lower incidence of adverse effects compared to synthetic products [13]. In this context, various substances, such as honey, have been investigated as potential active ingredients in cosmetic formulations. Therefore, the objective of this study was to systematically review published articles evaluating the efficacy of honey as a moisturizing cosmetic ingredient in clinical trials.

## 2 Methods

The systematic review was conducted in accordance with the guidelines of the PRISMA (Preferred Reporting Items for Systematic Reviews and Meta‐Analyses) statement [14].

### 2.1 Research Question

The present study evaluated the effects of cosmetics and dermocosmetics formulated with honey, applied to human subjects, including only randomized clinical trials that incorporated control groups and conducted efficacy testing. Considering that some clinical trials evaluating topical honey formulations are conducted within therapeutic or wound‐care contexts, the outcomes identified in this review were organized into two analytical categories: Cosmetic/dermocosmetic outcomes and medical or wound‐healing outcomes. This approach allowed a clearer interpretation of the available clinical evidence while maintaining the focus on the potential dermatological and cosmetic relevance of honey‐containing formulations.

### 2.2 Search Strategy and Screening

A manual search was conducted across five electronic databases—Scopus, PubMed, Web of Science, Embase, and the Cochrane Library—up to April 2025. Keywords and their variations were extracted from Medical Subject Headings (MeSH) and Health Sciences Descriptors (DeCS), and were used to construct the search strategy: “honey,” “skin,” “dermis,” “epidermis,” “hydration,” “moisturization,” “moisturizing,” “water retention,” “efficacy,” “effectiveness,” “clinical effect,” and “outcome”. These terms were adjusted according to the indexing rules and syntax of each database.

After removal of duplicates, the remaining records were screened based on titles and abstracts, applying predefined inclusion and exclusion criteria. During the full‐text assessment stage, three studies were excluded due to the absence of control groups, and two because the evaluated formulations did not contain apicultural products as primary components. Consequently, five studies met all inclusion criteria and were incorporated into the qualitative synthesis.

### 2.3 Inclusion and Exclusion Criteria

The inclusion criteria were: (1) use of honey in topical formulations, (2) randomized clinical trials, (3) presence of both positive and negative control groups (isolated patients or test areas), and (4) efficacy trials reporting outcomes related to skin improvement (such as wound healing, hydration, among others).

The exclusion criteria included: (1) reviews, articles without clinical trials, animal studies, case reports without clinical application, conference abstracts, opinion pieces, patents, studies not primarily focused on honey as a cosmetic ingredient; (2) studies lacking control groups; (3) studies involving patients undergoing aesthetic procedures; and (4) studies without documented ethical approval.

### 2.4 Data Extraction and Synthesis

Full texts of eligible studies were reviewed and essential data extracted, including author, year of publication, title, demographic information, honey concentration when reported, outcomes, and conclusions. The rationale for data selection was based primarily on the effects of honey as a cosmetic ingredient, variations in its use, and consequent impacts on formulation and skin response.

### 2.5 Risk of Bias Assessment

The methodological quality of the included randomized clinical trials was evaluated qualitatively by examining key aspects such as study design, presence of control groups, sample size, clarity of randomization procedures, and transparency in outcome reporting. Given the limited number of eligible studies and the variability in experimental designs and outcome measures, a formal quantitative risk‐of‐bias scoring tool was not applied. Nevertheless, the methodological characteristics of each study were carefully considered during the interpretation of the findings.

## 3 Results

### 3.1 Search and Screening

**FIGURE 1:**
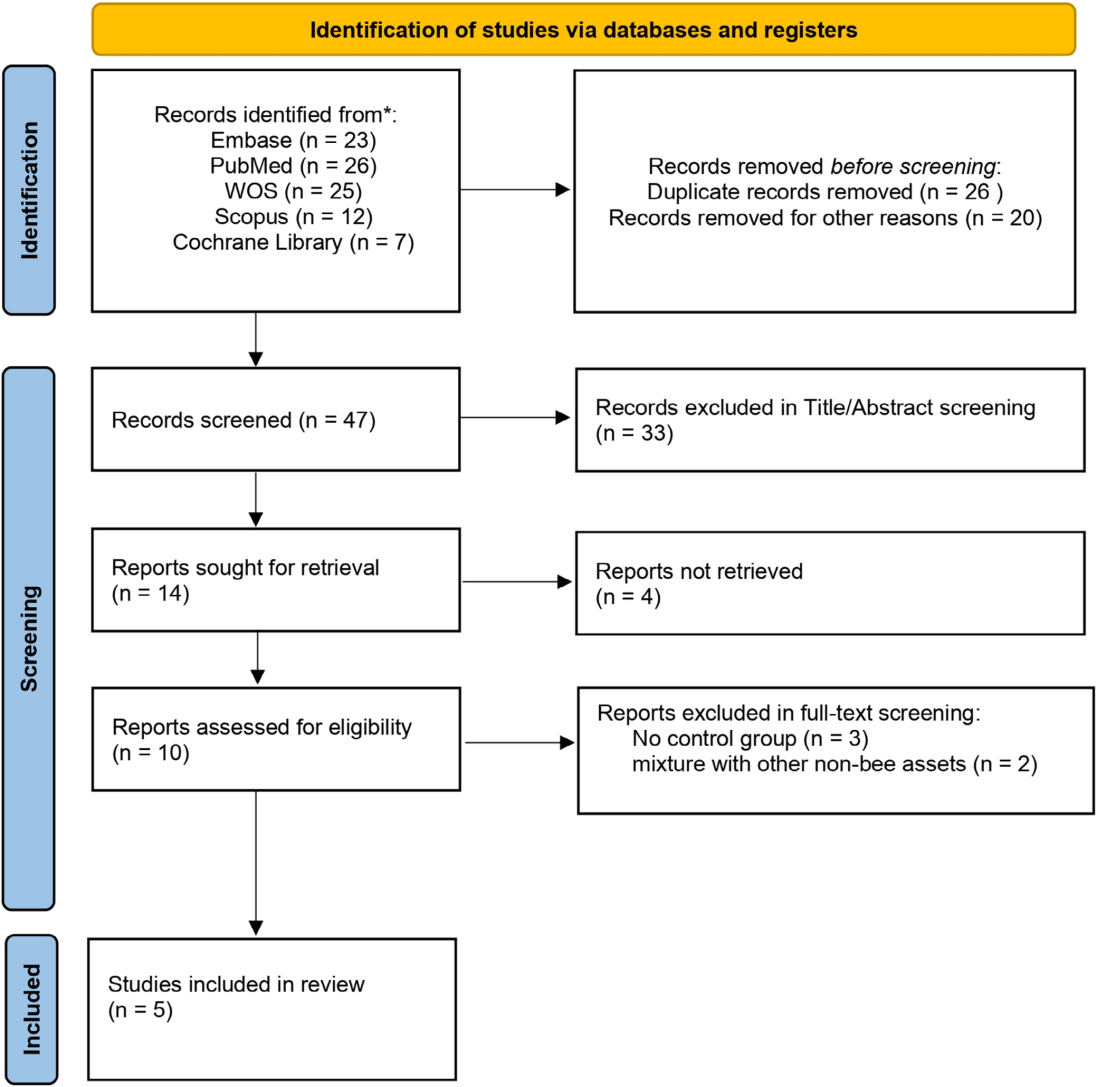
PRISMA flow diagram of literature search and study selection.

The initial search yielded 93 records. After removing duplicates and ineligible documents, 47 studies remained for screening. Title and abstract screening excluded 33 studies, resulting in 14 articles. Of these, four full texts could not be retrieved, leaving 10 for full‐text review. During full‐text screening, three studies were excluded due to the absence of control groups, and two because the evaluated formulations did not contain apicultural product bases. Ultimately, five studies were included in the systematic review (Figure 1). The included studies differed in several aspects, including the botanical origin of honey, formulation composition, concentration of the active ingredient, study population, and evaluated skin parameters. Such variability reflects the diversity of experimental approaches used to investigate honey‐based formulations and highlights the need for careful qualitative interpretation of the results.

### 3.2 Cosmetic and Dermocosmetic Outcomes

Two studies evaluated honey‐containing cosmetic formulations focusing on skin hydration and barrier function. Pavlačková et al. (2020) investigated cosmetic matrices containing honey and other bee products and reported improved moisturizing and barrier properties, particularly in formulations containing glycerinated honey extract. Similarly, Suwinski and Nowak (2024) evaluated hand creams containing multifloral honey in different concentrations and observed significant improvements in skin hydration, smoothness, and wrinkle parameters after four weeks of application. These studies specifically addressed cosmetic or dermocosmetic outcomes related to skin hydration and barrier function [7].

### 3.3 Medical and Wound‐Healing Outcomes

Three studies investigated therapeutic applications of honey‐containing formulations in wound‐related conditions. Heidari et al. (2013) [15], evaluated the use of Astragalus gossypinus honey in cesarean wounds but did not observe significant differences compared with placebo. Similarly, White (2012) compared a manuka honey‐based post‐tattoo cream with a conventional aftercare product and found no statistically significant differences in healing outcomes. In contrast, Sankar et al. (2021) reported faster healing of pressure injuries in critically ill pediatric patients treated with medicated honey dressings compared with standard treatment. Although these studies primarily addressed medical or wound‐healing outcomes, they were included because they provide clinical evidence regarding the effects of topical honey formulations on skin response and repair.

### 3.4 Study Characteristics

Five studies were included in the systematic review. The studies reported varied responses to honey‐containing cosmetic formulations; three of the five studies demonstrated beneficial effects of honey, while two showed no significant differences compared to controls. No adverse effects were reported in any of the studies. The characteristics of the selected studies are summarized in Table 1.

**TABLE 1:** Summary of the characteristics of the included studies.

Author, year	Country	Type of honey/+ bee products	Title	Number of patients/gender	Control	Objective	Results	Conclusion
Heidari et al.,2013 [15]	Iran	Astragalus gossypinus Honey	Does Iranian Astragalus gossypinus honey assist in healing cesarean wounds and scars?	132 female participants aged 17–35 years, in a gestational period of 37 to 42 weeks	42 with placebo 46 with control	To evaluate the effectiveness, healing time, and prevention of scars in post‐operative women	The comparative medical evaluation between the two groups, 10 and 40 days postoperatively, were not significantly different.	The results do not support the use of Iranian *Astragalus gossypinus* honey to accelerate or prevent scarring.
Pavlačková et al., 2020 [16]	Czech Republic	Flower honey (5% and 10%), forest honey (5% and 10%), Aqua‐mel extract (2% and 10%), Glycerin–aqua–mel extract (2% and 10%), Propolis (1%), Beeswax (1% and 3%), and Royal jelly (0.5%)	Hydration and Barrier Potential of Cosmetic Matrices with Bee Products	24 women aged between 23 and 49 years old	Dorsal side of left forearm	To evaluate the moisturizing and barrier action of emulsion formulations with the addition of honey and bee products	Moisturizing properties were observed in formulations with glycerinated honey extract. Flower honey showed greater moisturizing action when compared to forest honey. And the moisturizing and barrier properties were improved with formulations containing glycerin and 10% aqueous honey extract.	Cosmetic formulations enriched with honey and bee products are suitable for skin frequently exposed to surfactants present in personal hygiene products and cleaning agents.
Suwinski & Nowak, 2024 [17]	Poland	Multifloral honey in concentrations of 5%, 10%, and 15%	Innovative Honey‐Based Product and Its Beneficial Effects Measured by Modern Biophysical and Imaging Skin Techniques	24/20 women and 4 men	A placebo group	To evaluate the effects of hand cream formulations with different concentrations of multifloral honey (0%, 5%, 10% and 15%) on skin parameters, as well as their sensory characteristics.	After 4 weeks of use, honey‐infused creams demonstrated significant improvements in skin hydration (up to 29.7%), smoothness (up to 21.3%), reduction in wrinkle area (up to 21.4%), and average decrease in wrinkle depth (up to 11.7%). Among the sets of parameters evaluated, it was found that honey formulations were effective. In particular, the 5% concentration proved to be more effective in moisturizing, smoothing, and reducing wrinkles.	The study demonstrated the effectiveness of hand creams enriched with honey in improving the appearance of the skin, as well as providing favorable sensory aspects. The findings reinforce the use of this honey for dermatological formulations, in addition to offering support for overcoming the challenges associated with incorporating honey into cosmetics.
White, 2012 [18]	United Kingdom	Manuka honey UMF 10+	Tattoos as wounds: A clinical efficacy study of two skin aftercare preparations	The research began with 31 participants (13 men aged 18 to 47 and 18 women aged 19 to 42) and was completed with 25 (11 men aged 18 to 42 and 14 women aged 19 to 42).	Application of parallel control to the product with the asset	Comparing the cream formulated with manuka honey with the usual post‐tattoo cream in skin recovery levels	The results did not indicate statistically significant differences between the two products in relation to any of the parameters analyzed.	It was not possible to prove a significant clinical advantage for the cream formulated with manuka honey. However, the study confirms the viability of using both products for the recovery of post‐tattooed skin, with efficacy in the function and appearance of the skin.
Sankar et al., 2021 [19]	India	Not informed	Use of Honey Vs. Standard Care for Hospital‐Acquired Pressure Injury in Critically Ill Children: A Multicenter Randomized Controlled Trial	99 boys	48—Standard hospital treatment group	To evaluate whether medicated honey in dressings is more effective than standard treatment in the complete healing process of pressure injuries in children admitted to the Pediatric Intensive Care Unit.	Pressure injuries treated with honey dressings took an average of 7 days to heal completely (95% CI: 6–7 days), compared with 9 days with standard dressings (95% CI: 7–10 days). The honey group was approximately 1.9 times more likely to heal than the control group. No allergic reactions or secondary infections were observed in the honey group.	The use of medicated honey dressings significantly reduced the total healing time of wounds. Furthermore, the absence of allergic reactions to the dressings may indicate this as a new method of maintaining pressure injuries.

All studies reported on the use of honey in topical formulations. When reported, honey concentration showed efficacy in one study where a 10% glycerinated honey extract emulsion exhibited a progressive moisturizing effect [16]. In another study, a 5% concentration of raw honey in the formulation was effective in improving skin hydration, smoothness, and wrinkle reduction [17]. Both studies highlight honey as an effective moisturizing agent, noting benefits such as formulation stability over time [16] and suitability for skincare, especially for skin repeatedly exposed to cosmetic products containing surfactants [16].

Another study with positive outcomes reported significant results in a honey‐based formulation for wound healing in critically ill pediatric patients in intensive care units, demonstrating a faster healing time compared to the standard treatment [19]. The absence of allergic reactions or secondary bacterial infections was also noted.

In contrast, both the study by Heidari et al. (2013) [15], which evaluated efficacy, prevention of scar development, healing time, and scar grade with a product enriched with honey from Astragalus gossypinus in women post‐cesarean section, and the study by White (2012) [16], which compared the effects of a new post‐tattoo cream with a control ointment on recently tattooed skin using Manuka honey UMF 10+, found no significant results. Concentration data of the honey used in these latter studies was not provided.

## 4 Discussion

The data obtained from the analyzed studies demonstrate that some honey‐containing formulations have moisturizing, softening, wrinkle‐reducing, and healing activities. These specific studies did not indicate the floral source or perform melissopalynological analysis, except one that designated flower honey and forest honey without more specific information about the botanical origin. On the other hand, formulations with manuka honey and Astragalus gossypinus honey were not effective. These findings raise an important question regarding cosmetic formulations that use honey as an active ingredient: Whether botanical origin influences the observed effects. The available evidence suggests that botanical origin may influence the physicochemical properties of honey, especially when related to the levels of reducing sugars present in this product [20]; however, clinical evidence remains limited, and further controlled studies are necessary to confirm this relationship. Although these observations suggest that the botanical origin of honey may influence its dermatological performance, the current body of clinical evidence remains relatively limited. Therefore, further controlled clinical investigations are necessary to clarify the extent to which compositional variations contribute to differences in skin‐related outcomes.

For example, the study by Pavlackova et al. (2020) [16] indicated a 15% higher hydration index for flower honey, which has a slightly higher content of reducing sugars compared to forest honey. This supports the relationship with skin hydration, since the free hydroxyl groups of these sugars allow the formation of hydrogen bonds with water, increasing the moisture of the stratum corneum and promoting skin hydration [17]. Thus, in a general sense, honeys with higher sugar contents may be more effective, even at lower concentrations in cosmetics, helping to overcome challenges related to the viscosity and stickiness of products.

The set of substances present in honey is responsible for hydration both directly, such as sugars, and indirectly, such as amino acids [21], proteins [22], organic acids [23], vitamins [24], minerals [25], and phytoconstituents [26, 27, 28]. They act similarly, with an emphasis on hydroxyl‐dependent reactions as mentioned above. Moreover, some constituents are responsible for nutritive, antimicrobial, humectant, and antioxidant actions, making honey a key product for cosmetic research and development [5].

For centuries, honey has played an important role in traditional medicine; its uses for treating wounds and burns were key to establishing the product for therapeutic uses [29, 30, 31]. In vitro studies evaluated the potential of acacia, buckwheat, and manuka honeys in wound repair using human dermal fibroblasts. The results showed that acacia and buckwheat honeys were more effective in the healing process compared to manuka honey, suggesting greater efficacy in wound healing [32]. This aligns with White's (2012) [18] study, where the tested product containing manuka honey did not show significant differences compared to the traditional ointment.

In a randomized clinical trial conducted by Ahangar et al. (2023) [33] on the use of honey in skin graft fixation compared to the standard method, better results were achieved with honey, reducing adverse effects and treatment time. Another study showed that honey‐impregnated gauzes had a faster effect on re‐epithelialization, in addition to being effective, safe, and practical [34]. Similar results were presented by Sankar et al. (2021) [19], who obtained better results with honey‐containing formulations, with a faster healing period compared to the standard treatment. This may relate to the studies showing honey's effectiveness in skin hydration [16, 17], since better hydration improves the healing process.

Regarding floral origin and skin effects, studies indicate that differences exist. The eudermic properties of five citrus honeys, one acacia, one chestnut, and one multifloral honey showed that *Citrus* 1 and 3 samples and chestnut honey had the best results in cell viability and collagenase inhibition, with chestnut honey also standing out for its high polyphenol content and strong antioxidant activity [35]. Another study evaluated linden, sunflower, simple multifloral, two mountain multiflorals, meadow multifloral, and honeydew honeys regarding their healing power. The data showed that honeydew, one mountain multifloral sample, and meadow multifloral honey had the best results in stimulating fibroblast proliferation, keratinocyte migration, and collagen synthesis in vitro [36].

The data from this work support honey as a cosmetic ingredient, based on its benefits, especially regarding skin hydration and healing. However, the variation in its composition as well as the botanical origin directly influences the final product's results.

### 4.1 Study Limitations

This systematic review has some limitations that should be considered when interpreting the findings. First, only five randomized clinical trials met the inclusion criteria. Although this number is limited, these studies provide valuable clinical evidence regarding the topical use of honey in skin‐related applications. Second, the included studies presented heterogeneity in terms of honey type, botanical origin, formulation, concentration, study population, and evaluated outcomes. Due to this variability, a quantitative synthesis such as meta‐analysis was not feasible; however, the qualitative analysis allowed identification of relevant patterns and trends in the reported results. Finally, some studies investigated therapeutic or wound‐healing applications rather than strictly cosmetic outcomes. Nevertheless, these studies were considered relevant because they provide clinical insight into the biological effects of honey on skin response and repair, which are closely related to dermocosmetic applications.

## 5 Conclusion

Honey‐based cosmetics have shown divergent results regarding their therapeutic effects on skin hydration and wound healing; however, promising clinical outcomes were observed in the evaluated studies. Especially in clinical trials, the ingredient demonstrated significant improvement compared to control groups and standard therapy groups, making it a promising ingredient for the cosmetic industry. Nevertheless, it is important to note that studies in this area are still scarce, and evaluation methods are not yet standardized across trials, which hampers the clarity of the findings. However, the limited number of randomized clinical trials and the heterogeneity among the included studies restrict the strength of these conclusions. Additional well‐designed clinical trials focusing specifically on cosmetic outcomes are necessary to better establish the role of honey as an active ingredient in dermocosmetic formulations. Indications that botanical origin may influence the final product are likely related to its chemical composition, such as the percentage of reducing sugars and other metabolites.

## Conflicts of Interest

The authors declare no conflicts of interest.

## Data Availability

Data sharing not applicable to this article as no datasets were generated or analysed during the current study.
